# High Plasma Lipid Levels Reduce Efficacy of Adenovirus-Mediated Gene Therapy

**DOI:** 10.1038/s41598-017-00376-5

**Published:** 2017-03-24

**Authors:** A. M. Kivelä, J. Huusko, E. Gurzeler, A. Laine, M. H. Dijkstra, G. Dragneva, C. B. F. Andersen, S. K. Moestrup, S. Ylä-Herttuala

**Affiliations:** 1 University of Eastern Finland, A.I. Virtanen Institute for Molecular Sciences, Department of Biotechnology and Molecular Medicine, P.O. Box 1627, FIN-70211 Kuopio, Finland; 2 University of Aarhus, Department of Biomedicine, Ole Worms Allé 3, DK-8000 Aarhus C, Denmark

## Abstract

Adenoviruses are very efficient vectors for delivering therapeutic genes in preclinical and clinical trials. However, randomized controlled human trials have often been lacking clear clinically relevant results. We hypothesized that high lipid levels and specific lipoproteins could significantly decrease adenoviral transduction efficiency *in vivo*. Here we demonstrate that mice on a high fat diet have lower transgene expression compared to mice on a regular chow. In addition, on a high fat diet, ApoE^−/−^ mice have much higher plasma transgene levels compared to LDLR-deficient mice. We also found that specific lipoprotein receptors play an important role in adenoviral transduction. These findings suggest that high plasma lipid levels, especially apoE-containing lipoproteins, reduce efficacy of adenoviral transduction in mice, which implies that high cholesterol levels in humans could be protective against viral infections and also lead to insufficient transgene expression in clinical trials using adenoviral vectors.

## Introduction

Replication deficient adenoviruses are widely used and currently the most efficient vectors for delivering therapeutic genes in preclinical and clinical studies^1, 2^. In the field of cardiovascular gene therapy, the results from preclinical trials have been promising and several factors with therapeutic potential have been identified. In cardiovascular gene therapy clinical trials, adenoviral vectors have been used to treat coronary artery disease, peripheral artery disease and heart failure. In these trials, vectors have been directed to ischemic or damaged myocardium, ischemic skeletal muscle or vascular wall with the concept of therapeutic angiogenesis or improved contractility. The overall safety of adenoviruses has been very good, symptoms of common cold being the most widely reported side-effect. However, promising results from preclinical studies have not been translated to clinical benefits since none of the randomized controlled trials have shown clear clinically relevant results^1, 2^.

One likely explanation for the lack of therapeutic effects in clinical trials is that sufficient levels of transgene expression in target tissues have not been reached. In preclinical settings, adenoviral vector DNA has been shown to be expressed in several different tissues^3^. Liver is the most prominent organ for the transduction: after intravenous administration, most of the adenovirus enters in the liver. Coxsackie-and-adenovirus receptor (CAR) has been shown to play a crucial role in the adhesion and uptake of adenovirus *in vitro* ^4^. However, Shayakhmetov *et al*. have reported a novel CAR-independent pathway used by adenoviruses for the transduction of hepatocytes *in vivo* ^5^. In this pathway, viral particles first bind to blood coagulation factor FIX and the complement factor C4BP which enable the binding of these complexes to heparan sulfate proteoglycans (HSPG) and LDL receptor related protein (LRP) leading to internalization of the viruses. These kind of interactions have also been reported with several other viruses. For example, LDL receptor has been shown to serve as a receptor for rhinovirus and hepatitis C virus^6, 7^. Therefore, it has been suggested that lipoproteins could participate in the innate immunity not only via direct neutralization of viruses but also via competition with viral uptake into the cells^8^.

We previously reported differences in plasma transgene expression after systemic adenoviral (Ad) human VEGF-A gene transfer in four different hyperlipidemic mouse models although the same amount of virus was used in all models^9^. Since significant differences in lipoprotein metabolism exist between different hyperlipidemic mouse models, it is possible that not only the increased lipid levels but also specific lipoproteins could compete with the virus uptake via lipoprotein receptors leading to significant differences in the transduction efficiency in gene transfer studies.

In the present study, we compared the efficacy of AdVEGF-A gene transfer in C57Bl/6j, LDLR^−/−^, LDLR^−/−^ApoB^100/100^, ApoE^−/−^ and LDLR^−/−^ApoE^−/−^ mice in order to find out whether Ad transduction could be affected by lipoproteins and lipoprotein receptors. We demonstrate that mice fed with a high fat diet (HFD) had a significantly lower transgene expression after both systemic and local AdVEGF-A gene transfer compared to mice on a regular chow diet (RCD) indicating that higher lipid levels are decreasing Ad transduction efficiency. More specifically, on a HFD apoE-containing lipoproteins had a major role in the reduction of Ad transduction efficacy in mice. Lipoproteins inhibited Ad uptake via a pathway mediated by HSPGs, LRP and LDLR. Together, these findings suggest that high lipid levels also in humans could affect viral transduction leading to insufficient transgene expression after Ad gene transfer. These results might partly explain the disappointing results after cardiovascular clinical Ad gene therapy trials as the treated patients in most cases have had significantly elevated blood lipid levels.

## Results

### High lipid levels inhibit adenoviral transduction

**Figure 1:**
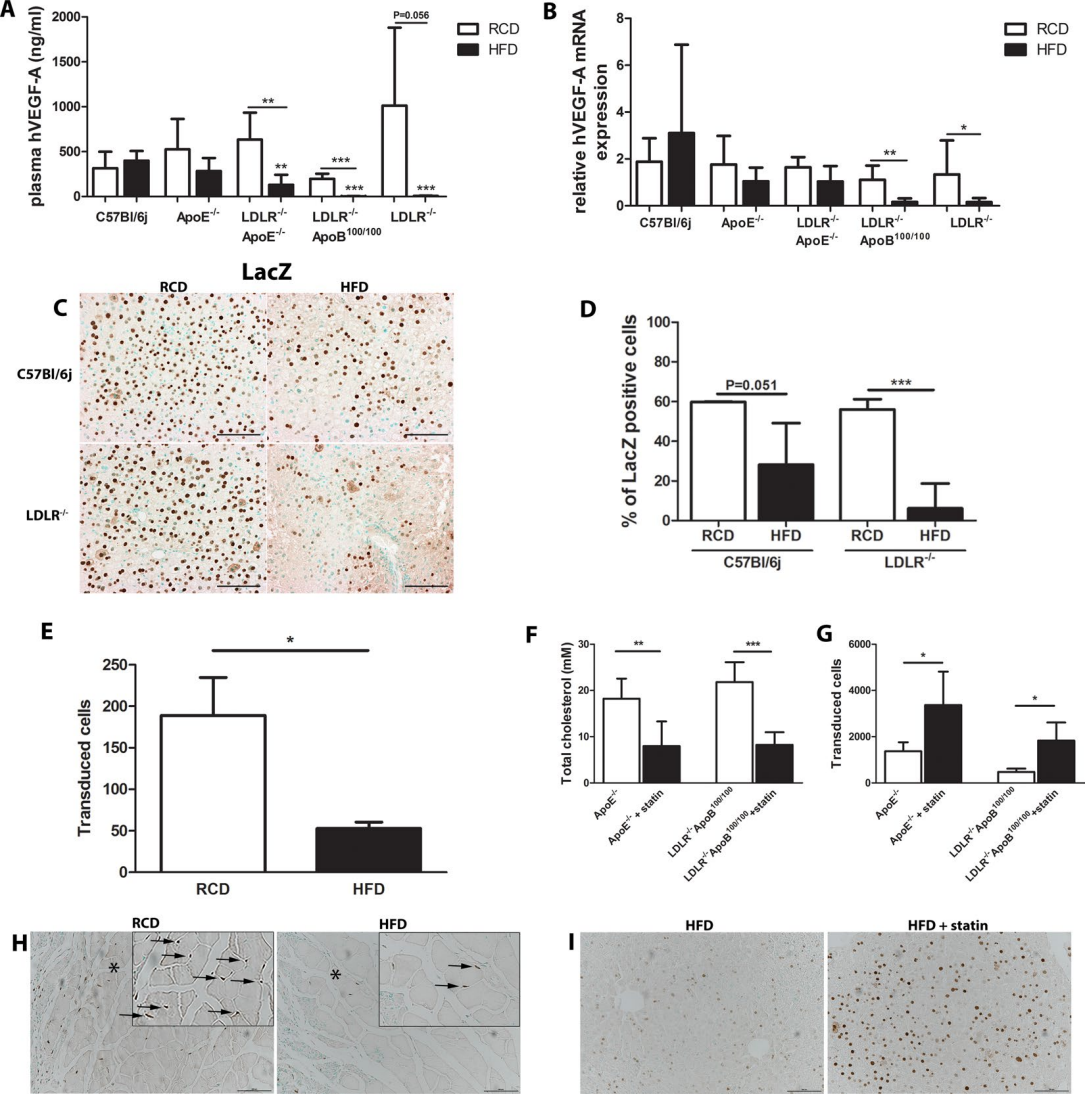
High lipid levels inhibit adenovirus-mediated transgene expression. Plasma hVEGF-A protein expression analyzed by ELISA (**A**) and liver hVEGF-A mRNA expression analyzed by qRT-PCR (**B**) in different mouse strains either on a RCD or HFD four days after systemic AdVEGF-A gene transfer. N = 5–8 per group. To verify that this finding was not transgene-dependent, transduction efficiency was studied also using AdLacZ and a similar inhibitory effect in transgene expression was seen when animals were on a HFD (**C**, left panel pictures vs. right panel pictures). The number of LacZ positive cells was significantly lower in LDLR^−/−^ mice on a HFD compared to mice on a RCD (**D**). Representative pictures of LacZ staining in (**C**). Scale bars 100 µm. N = 4, cell numbers analysed from five microscopic fields/mouse (**D**). The decrease in the transduction efficiency was also seen after a local AdLacZ injection into skeletal muscles (posterior calf). The number of transduced cells being significantly higher in mice fed with RCD (**E**,**H**, left picture) compared to mice fed with HFD (**E**,**H**, right picture). Examples of positive staining are highlighted with arrows in greater magnification inserts from site indicated with asterisk (**H**). Statin treatment significantly lowered total cholesterol in ApoE^−/−^ and LDLR^−/−^ApoB^100/100^ mice (**F**) and this was further seen as a significant increase in the amount of transduced hepatocytes four days after systemic AdLacZ gene transfer (**G**,**I**). Representative pictures of LacZ staining in H and I. Scale bars 100 µm, N = 6.

To assess whether high lipid levels affect Ad transduction efficiency in the liver, transgene expression after systemic gene transfers of AdhVEGF-A and AdLacZ was compared in C57Bl/6j mice and four different hyperlipidemic mouse models (LDLR^−/−^, LDLR^−/−^ApoB^100/100^, ApoE^−/−^ and LDLR^−/−^ApoE^−/−^) fed either with a RCD or HFD. Plasma hVEGF-A levels were measured four days after gene transfer. On a RCD, hVEGF-A expression was extremely high in all hyperlipidemic models as well as in control C57Bl/6j mice and no significant differences were seen between the study groups (Fig. 1A, white bars). However, hyperlipidemic models on a HFD, in general, had a lower hVEGF-A expression compared to mice on a RCD (Fig. 1A). Also, in LDLR-deficient models, hVEGF-A expression was significantly lower compared to C57Bl/6j and ApoE^−/−^ mice (Fig. 1A). Similar results were seen when the liver hVEGF-A mRNA levels were compared between different groups with both diets (Fig. 1B). To verify that the differences in the transgene expression after Ad injections were not dependent on the transgene itself, the expression of beta-galactosidase protein was also compared between different groups with both diets four days after systemic AdLacZ injections (Fig. 1C,D). Similarly, beta-galactosidase expression was high in mice on a RCD (Fig. 1C, left panel pictures and 1D) whereas HFD decreased the expression (Fig. 1C, right panel pictures and D). In LDLR^−/−^ mice after a HFD beta-galactosidase expression was significantly decreased compared to mice on RCD (Fig. 1D).

In human clinical trials, local gene transfer is commonly performed instead of systemic gene transfer. Therefore, the effect of high lipid levels on Ad transduction efficiency was also studied locally four days after intra muscular (posterior calf) AdLacZ injections. Similarly, as was seen after systemic gene transfer in the liver, high lipid levels reduced Ad transduction locally in the skeletal muscle: in LDLR^−/−^ApoB^100/100^ mice on a HFD beta-galactosidase expression was significantly decreased compared to mice on a RCD (Fig. 1E,H).

Currently, statins are the most commonly used drugs for managing hypercholesterolemia in the treatment of cardiovascular diseases in humans. To see whether lipid lowering by statins could improve Ad transduction efficiency, ApoE^−/−^ and LDLR^−/−^ApoB^100/100^ mice were fed either with a control HFD (10 weeks) or with a HFD containing rosuvastatin (normal HFD 6 weeks + rosuvastatin HFD 4 weeks) and systemic AdLacZ injections were performed. Rosuvastatin decreased total cholesterol levels in both strains (Fig. 1F) and this decrease in cholesterol levels was associated with significantly higher beta-galactosidase expression in the liver (Fig. 1G,I).

### Differences in the transduction levels correspond to the biological effects of transgenes

**Figure 2:**
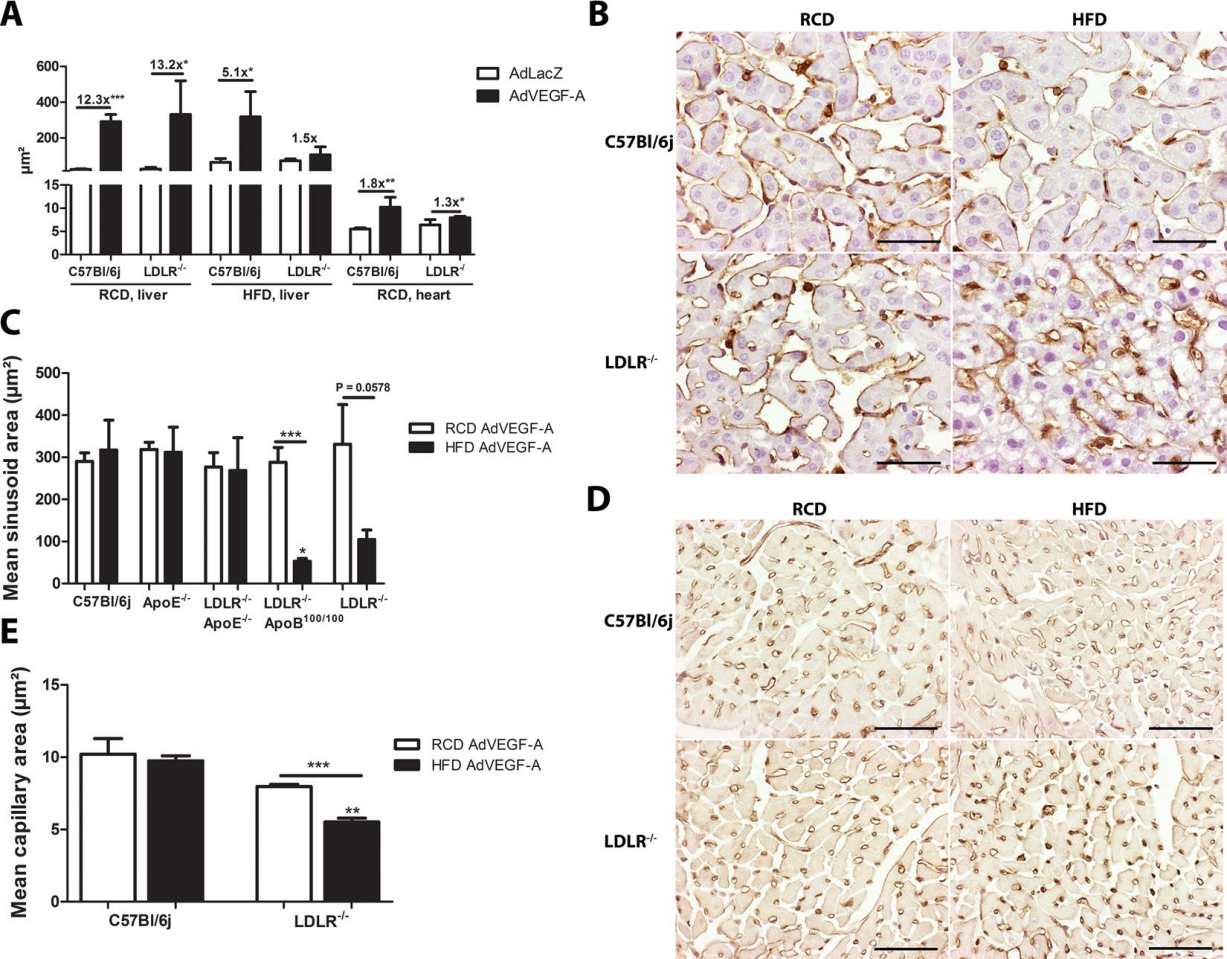
Differences in the transgene expression levels predict biological effects of the transgene. The biological effect of hVEGF-A was seen as enlarged sinusoid area in the liver (**A**,**B**,**C**) and capillary area in the heart (**A**,**D**,**E**). The decrease in the sinusoid area was significant after a HFD compared to RCD in LDLR^−/−^ApoB^100/100^ and LDLR^−/−^ mice (**C**) and in the heart capillary area in LDLR^−/−^ mice (**E**). Representative pictures from endothelium-stained sections of AdVEGF-A transduced livers (**B**) and heart (**D**). Scale bars 50 µm, N = 4, sinusoid and capillary area analyzed from five microscopic fields/mouse. Fold increase is shown above compared columns in panel A.

After intravenous administration, Ad is known to transduce mainly the liver^3^. VEGF-A is known to cause pronounced angiogenic effects seen as enlarged capillaries in the target tissue. Indeed, in this study, high hVEGF-A expression led to an expected angiogenic response most prominently in the liver, causing pathological dilation of the sinusoids (12.3 and 13.2 fold increase in AdhVEGF-A group vs. AdLacZ group in C57Bl/6j and LDLR^−/−^ mice, respectively, on a RCD, Fig. 2A) four days after the gene transfer. On a HFD the angiogenic effect of VEGF-A was clearly less pronounced (5.1 and 1.5 fold increase in AdhVEGF-A group vs. AdLacZ group in C57Bl/6j and LDLR^−/−^ mice, respectively, Fig. 2A) showing an inhibitory role of high blood lipids in the Ad transduction. Also, the importance of LDLR and the inhibitory effect of apoE-containing lipoproteins in the Ad transduction was highlighted when directly comparing the biological effect of AdVEGF-A in the different mouse strains (Fig. 2B,C). The angiogenic effect seen as dilation of liver sinusoids was decreased in LDLR^−/−^ApoB^100/100^ and LDLR^−/−^ mice fed on a HFD compared to the same strains on a RCD (Fig. 2B,C). However, when ApoE was absent (ApoE^−/−^ and LDLR^−/−^ApoE^−/−^ mice) HFD did not diminish the angiogenic effect of hVEGF-A (Fig. 2C). Also other tissues were affected by the AdVEGF-A gene transfer since capillary dilatation was also seen in the hearts of the mice four days after AdVEGF-A gene transfer (1.8 and 1.3 fold increase in AdhVEGF-A group vs. AdLacZ group in C57Bl/6j and LDLR^−/−^ mice, respectively, on a RCD, Fig. 2A). Again, the angiogenic response was milder in LDLR^−/−^ mice after a HFD with significantly smaller mean capillary area compared to LDLR^−/−^ mice on a RCD (Fig. 2D,E). Even though there was a clear biological effect of VEGF-A visible also in other tissues, there was no or very little measurable transgene mRNA expression in the heart or the lungs (Ct 35–38). Therefore, Ad transduction of other tissues after systemic gene transfer seems to be really low and the observed biological effect of hVEGF-A is more likely caused by circulating hVEGF-A produced in the liver.

### HSPG, LRP and LDLR are required for adenoviral transduction *in vivo*

**Figure 3:**
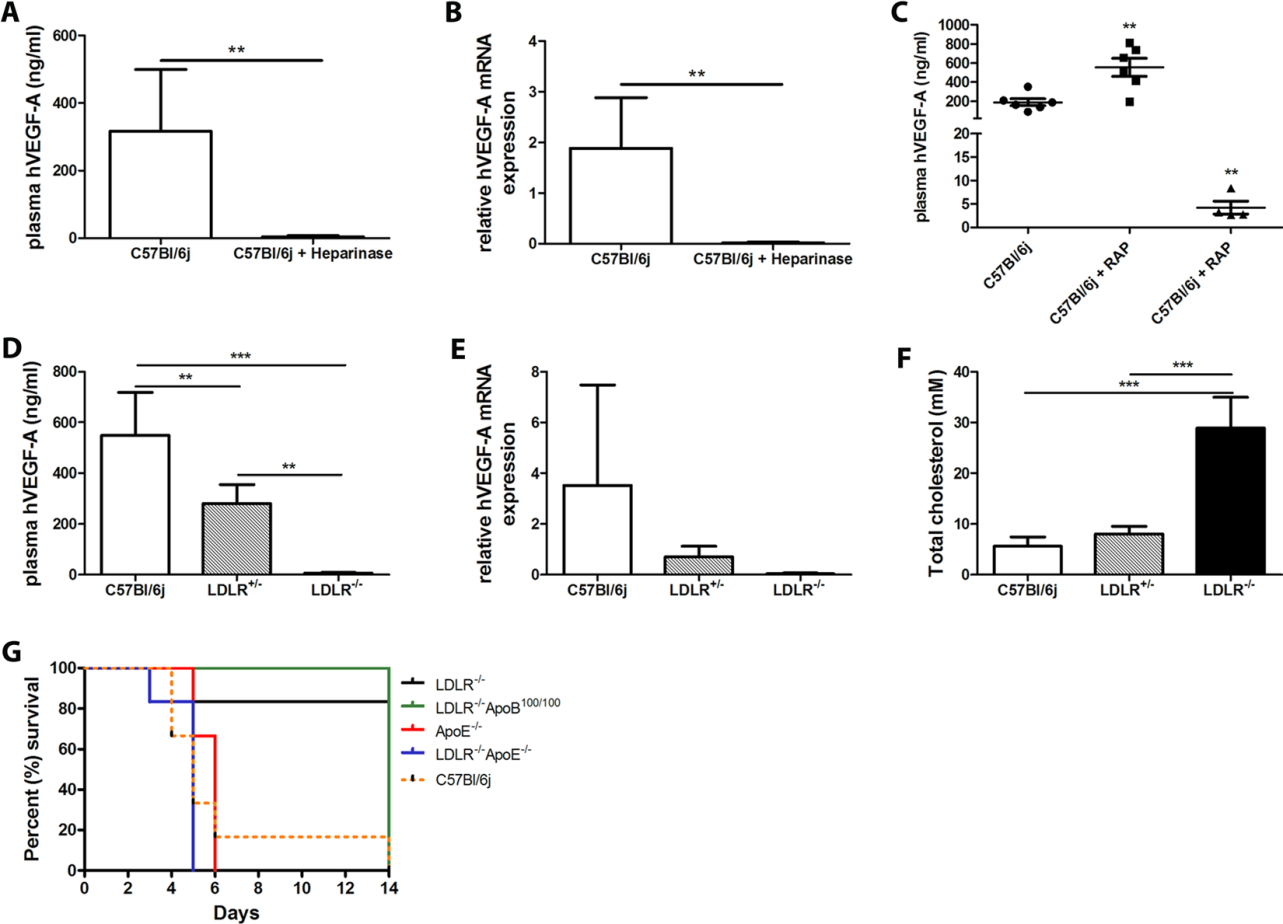
HSPG, LRP and LDLR play an important role in the adenoviral transduction *in vivo*. Heparinase injection significantly reduced plasma hVEGF-A protein level (**A**) and mRNA level in the liver (**B**). A specific LRP inhibitor, RAP, either increased or decreased plasma hVEGF-A level (**C**) most probably depending on the interaction preferences of RAP with either adenovirus or LRP. LDL receptor had a significant role in the uptake of adenovirus into the cells since mice having both LDLR alleles (C57Bl/6j) had significantly higher hVEGF-A protein levels after AdVEGF-A gene transfer compared to mice having only one (LDLR^+/−^) or no (LDLR^−/−^) alleles (**D**). Similar findings were seen in liver hVEGF-A mRNA levels (**E**). Wildtype (C57Bl/6j) and LDLR^+/−^ mice had similarly low total cholesterol levels which were significantly elevated in LDLR^−/−^ mice (**F**). Extremely high hVEGF-A levels in plasma contributed to decreased survival of the mice while increased lipid levels (animals on a HFD) and absence of LDLR improved the survival probably by competing with the virus entry into the cells (**G**). N = 5–8 per group.

Liver expresses high numbers of lipoprotein receptors and they may, together with HSPGs, play a role in Ad uptake into the cells. In order to find out the receptors, which mediate Ad uptake into the cells, C57Bl/6j mice on a RCD were first injected with heparinase 30 min before gene transfers. Heparinase degrades heparin and heparan sulfates reducing clearance of proteins whose catabolism is dependent of HSPGs^10^. Heparinase significantly reduced plasma hVEGF-A expression (Fig. 3A) as well as liver hVEGF-A mRNA (Fig. 3B) four days after gene transfer most probably due to reduced virus entry into the cells. LRP is a multifunctional receptor involved in lipoprotein metabolism and atherogenesis^11, 12^. Therefore, we wanted to study the role of LRP more specifically. C57Bl/6j mice on a RCD were injected with receptor-associated protein (RAP) 30 min before gene transfers. RAP is a common chaperone of all LDLR family members and when added exogenously it can block ligand binding to all LDLR family members except LDLR itself^13^. Surprisingly, RAP significantly either increased or robustly decreased plasma hVEGF-A expression three days after the gene transfer (Fig. 3C, Supplementary Table S1). The increased transgene expression could be explained by direct interaction of RAP and Ad before receptor binding since it has been shown that Ad can be targeted to LRP by chemical modification of the Ad pIX capsomere with RAP leading to increased transduction^14^. Therefore, we conducted an experiment where Ad was preincubated with RAP for 30 min before gene transfers, to see whether transgene expression could be affected due to possible interaction. Interestingly, already two days after gene transfer, plasma hVEGF-A levels were 13.0-fold higher than in control mice without RAP (p < 0.001) and 4.4-fold higher than in mice with RAP injection 30 min before gene transfer (p < 0.01) (Supplementary Table S1). This suggests that RAP is capable of interacting with Ad and target the complex rapidly and efficiently to the receptors needed for Ad internalization.

The role of LDLR in the virus uptake was studied using LDLR^−/−^ mice and mice either with one (LDLR^+/−^) or two alleles (C57Bl/6j) of LDLR on a HFD. LDLR^+/−^ mice express approximately half of the LDLR levels of normal mice with two receptor alleles^15^. When plasma hVEGF-A protein expression was measured four days after AdVEGF-A gene transfer, LDLR^+/−^ mice had significantly reduced amount of hVEGF-A compared to C57Bl/6j mice but also significantly higher hVEGF-A expression than LDLR^−/−^ mice (Fig. 3D). Similar results were seen when hVEGF-A mRNA expression was measured in the liver four days after gene transfer (Fig. 3E). Interestingly, even though LDLR^+/−^ mice have only half of the LDLR expression compared to C57Bl/6j mice, these both strains have equally low cholesterol levels after five weeks of HFD compared to LDLR^−/−^ mice with significantly elevated cholesterol levels on a HFD (Fig. 3F). Therefore, reduced transgene expression in LDLR^−/−^ mice on a HFD is not only due to elevated lipid levels but also the absence of LDLR.

The high VEGF-A expression in some strains not only led to an adverse angiogenic response in the liver but also decreased survival of these animals (Fig. 3G). 14 days after a high dose AdVEGF-A gene transfer 83% of LDLR^−/−^ and 100% of LDLR^−/−^ApoB^100/100^ mice (n = 6) on a HFD were alive whereas in other strains (n = 6 per strain) less than 20% survived (Fig. 3G). Plasma levels of hVEGF-A rising over 10 ng/ml started to be lethal. When animals with high plasma concentration of hVEGF-A were sacrificed, there was no macroscopically visible reason explaining the poor survival of the animals. Also, basic clinical chemistry showed no obvious reason for lethality. However, histological examination of the major organs revealed non-physiological, aberrantly enlarged sinusoid structures (Fig. 2A) correlating with the poor survival.

## Discussion

The goal of this study was to elucidate to effects of lipoprotein receptors and lipid levels on Ad transduction *in vivo* in order to find out whether high lipid levels and specific lipoproteins could affect Ad transduction efficiency and potentially relate to insufficient transgene expression in clinical trials. Here, we demonstrate significant differences in the adenoviral transduction efficiency after systemic VEGF-A gene transfer in four hyperlipidemic mouse models: LDLR^−/−^, LDLR^−/−^ApoB^100/100^, ApoE^−/−^ and LDLR^−/−^ApoE^−/−^ mice. In general, mice fed with the HFD have lower transgene expression compared to mice on the RCD. In addition, specific lipoproteins seem to have an effect on Ad transduction since on a HFD, transgene expression is reduced in apoE-deficient models compared to C57Bl/6j mice and even further reduced in LDLR^−/−^ and LDLR^−/−^ApoB^100/100^ mice. This clearly implies that high plasma lipid levels, especially apoE-containing lipoproteins, reduce Ad transduction efficacy in mice. In addition, lipoprotein receptors seem to play an important role in the uptake of Ad into the liver cells.

**Figure 4:**
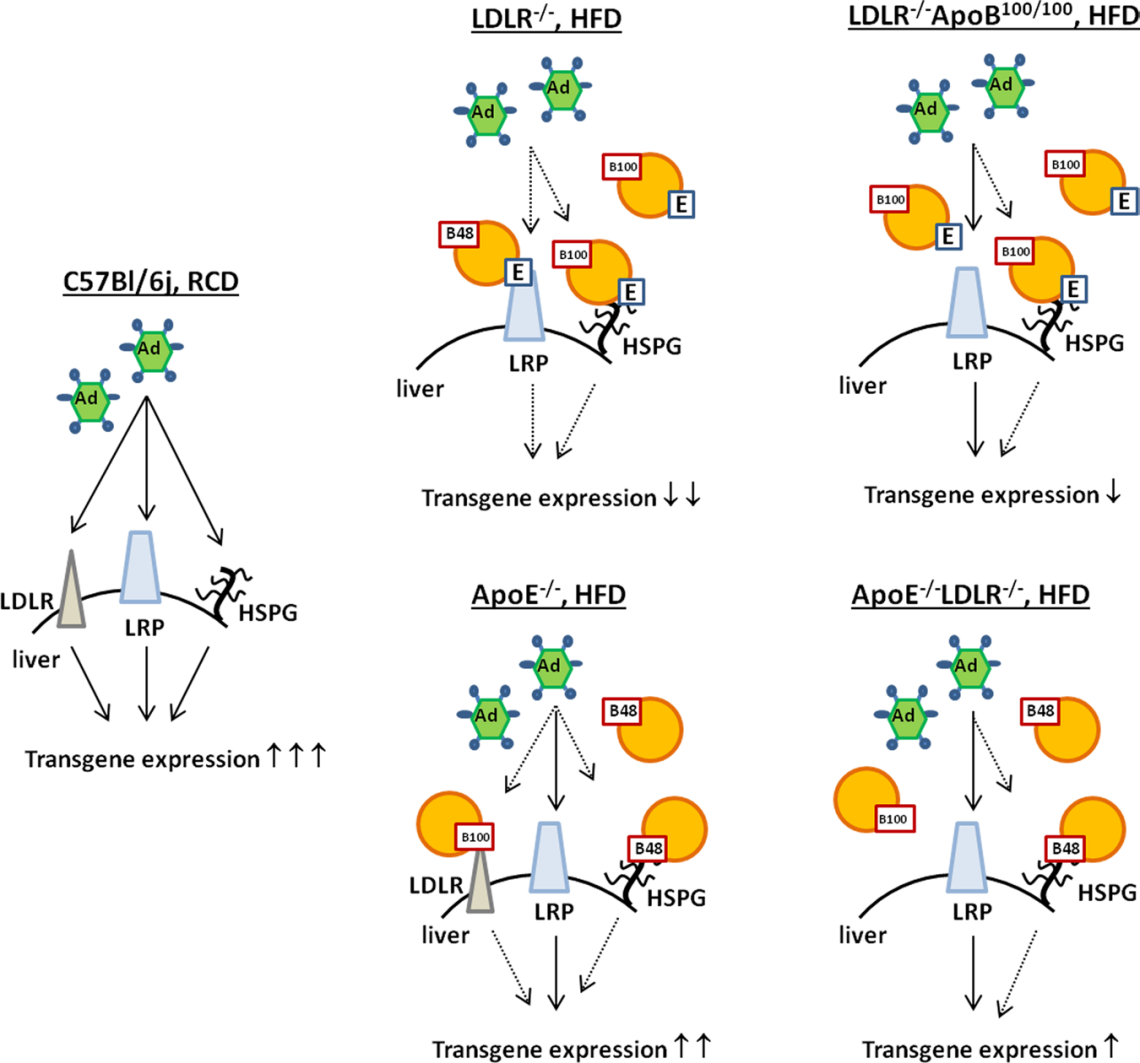
Proposed mechanism on how lipoproteins and lipoprotein receptors are affecting Ad transduction in different hyperlipidemic mouse models. There are three possible routes for Ad entry into the cells: LDLR, LRP and HSPG. High lipoprotein levels in hyperlipidemic mice on a HFD are competing with the Ad uptake via these different routes leading to a decreased transgene expression compared to C57Bl/6j mice. In apoE-deficient mice this competition with Ad uptake is not as efficient as in LDLR-deficient mice due to absence of apoE which mediates lipoprotein binding to LRP.

Most likely, high lipoprotein levels in mice are competing with the Ad uptake in the liver. It is possible that Ad uses LRP and HSPGs with the help of different blood factors for binding and internalization into the cells^16^. Our results suggest that the role of LDL receptor itself in the Ad uptake cannot be dismissed. LDLR^+/−^ mice that have approximately half of the LDLR expression compared to wild-type mice had significantly reduced plasma levels after AdVEGF-A gene transfer compared to C57Bl/6j mice even though these both strains have similar plasma lipid levels after five weeks on a HFD. This suggests that reduced amount of LDLR itself and not only the elevated lipid levels affect Ad transduction possibly via LDLR which serves as one route for virus entry into the cells (Fig. 4).

Shayakhmetov *et al*. excluded the involvement of apoE-containing lipoprotein particles in Ad transduction since LDLR^−/−^ApoE^−/−^ mice did not have further reduced Ad transduction compared to LDLR^−/−^ mice^5^. However, when apoE is absent, lipoprotein particles are not able to bind to LRP and therefore they do not efficiently compete with Ad in the cell uptake. Therefore, it is expected that Ad transduction is not further reduced in LDLR^−/−^ApoE^−/−^ mice. However, in the current study, when mice were challenged with a HFD, the role of apoE-containing lipoproteins became critical. LDLR^−/−^ mice had a significantly lower transgene expression after AdVEGF-A gene transfer compared to LDLR^−/−^ApoE^−/−^ mice. This is not surprising since LDLR is mediating the uptake of apoE-containing particles together with LRP. When LDLR is absent, LRP becomes the main receptor taking up apoE-containing particles in the liver and most probably leads to a more efficient competition with Ad (Fig. 4.). Therefore, in the light of the present results, comparing the effects of the same dose of Ad gene transfer in different hyperlipidemic mouse models is challenging. To be able to do a comparison between biological effects in different models, the levels of the therapeutic protein should be adjusted to the similar level instead of using the same dose of the vector.

This study has also limitations: First, lipoprotein metabolism in mice differs from that in humans. Wild type mice do not express CETP and approximately 70% of the VLDL secreted from the liver contains apoB-48 instead of apoB-100^17^. In this study, LDLR^−/−^ApoB^100/100^ mice have lipoprotein profile that resembles that of humans in the best possible way; cholesterol is mostly carried in LDL particles, HDL cholesterol is low, and VLDL and its remnants have apoB-100 as the major apolipoprotein^18^. Interestingly, LDLR^−/−^ApoB^100/100^ mice are, in addition to LDLR^−/−^ mice, the model in which the high lipid levels have the strongest effect on Ad transduction. Therefore, in the future it would be interesting to study whether this correlation between transgene expression and plasma cholesterol or lipoprotein levels is present also in clinical trials using Ad. Second, lipids are not the only factors affecting the efficacy of Ad transduction. Peng *et al*. reported differences in the innate immune responses against replication-defective Ads between different mouse strains^19^ while in this study the background inbred mouse strain in all used models is C57Bl/6j, the most commonly used inbred laboratory mouse strain. Third, in this study we used intravenous Ad administration, whereas in clinical trials local administration is used more often. Compared to the liver, in other tissues like in the heart or skeletal muscle the amount and type of lipoprotein receptors is different and their role in Ad uptake may not be as critical as in the liver. However, quantitative monitoring of the transgene expression would be very important in every gene therapy study despite of the route of administration.

Taken together, it is likely that the inhibitory effect of high lipid levels on Ad transduction has contributed to the disappointing outcomes in clinical trials. Physiological LDL cholesterol level in humans is approximately 1.5 mmol/l, after which LDLR saturate and become downregulated and less efficient in the clearance of lipoprotein particles^20^. The so-called normal LDL cholesterol level in humans (3.0 mmol/l) is already twice as high as the physiological level. It may be that one evolutionary reason for the selection of higher cholesterol and LDL levels especially in the Northern areas might have been the protection against viral infections. Nowadays, with effective cholesterol lowering treatments it is possible to reach LDL cholesterol levels close to the physiological level. It would be important to take into account the effects of the strong LDLR upregulation by statins with additional effects of PCSK9 inhibitors. In fact, LDL receptor has also been shown to serve as a receptor for hepatitis C virus (HCV)^21^ and furthermore, it has been shown that PCSK9 is able to block infection by HCV *in vitro* ^22^. Therefore, the number and type of infections should be carefully monitored in clinical trials. On the other hand, in Ad clinical trials patients with elevated plasma cholesterol levels could be first treated with statins to reach lower cholesterol levels to ensure efficient expression of the transgenes.

## Methods

### Experimental design

In this study the effects of high plasma lipid levels as well as lipoprotein receptors on the uptake of Ad into liver after systemic Ad gene transfer were addressed. We used genetically modified mouse models with different modified lipid profiles, namely LDLR^−/−^, ApoE^−/−^, LDLR^−/−^ApoE^−/−^ and LDLR^−/−^ApoB^100/100^ in C57Bl/6j background. C57Bl/6j mice served as controls to measure the number of transduced cells, transgene expression at mRNA and protein levels and the biological effects of the transgene to assess the strain-specific differences in these variables.

All animal experiments and procedures used in this study were approved by the National Animal Experiment Board in Finland and carried out in accordance with the EU Act on the Protection of Animals Used for Scientific or Educational Purposes (497/2013) and The Finnish Act on Animal Experimentation. Animals were kept in standard housing conditions in The National Laboratory Animal Center of The University of Eastern Finland. Diet and water were provided ad libitum. Some groups of mice were fed with high-fat, high cholesterol diet (Harlan Teklad, 0.15% of calories from cholesterol) for five weeks and some groups also got statin treatment (rosuvastatin, 10 µg/g/day, Crestor®, AstraZeneca) administered with the HFD for four weeks.

Systemic gene transfers of AdhVEGF-A or AdLacZ were done via tail vein injection in 200 µl volume using a 30 G needle. Local gene transfers were done with a direct, slow injection of AdLacZ into the skeletal muscles of the posterior calf of the animal in 50 µl volume using a 30 G needle. For the gene transfers, the mice were gently anesthetized s.c. with medetomine (1 mg/kg, Domitor®, Pfizer) and ketamine (75 mg/kg, Ketalar®, Pfizer). A dose of 0.7 × 10^9^ viral particles was used per mouse. The success of the gene transfer was observed during the injection and verified by transgene expression in the plasma. Exclusion criteria was set to be no observed transgene expression in the plasma, but none of the mice needed to be excluded from the study. Mice from each strain were randomly assigned to the different diet and gene transfer groups and all data analysis was done in a blinded fashion.

### Adenoviral vectors

Human clinical grade first generation serotype 5 replication deficient (E1, partially E3 deleted) Ad were produced under GMP conditions in 293 cells and analyzed to be free from endotoxin and microbiological contaminants^23, 24^. All viral constructs were tested *in vitro* for their infectivity and potency by analyzing protein production using western blotting.

### RNA isolation and quantitative real-time PCR

RNA was isolated with TRI-reagent (Sigma Aldrich). 1 µg of total RNA was used for the cDNA synthesis using random hexamer primers (Promega) and RevertAid^TM^ reverse transcriptase (Fermentas). The relative expression levels of mRNAs encoding hVEGF-A and peptidylprolyl isomerase A (PPIA) in different tissue samples were measured according to manufacturer’s protocol with quantitative RT-PCR (StepOnePlus, Applied Biosystems) using specific Assays-on-Demand target mixes (Hs00900054_m1 and Hs99999904_m1, respectively; Applied Biosystems). The expression levels were normalized to PPIA.

### Protein extraction and enzyme-linked immonoassay

Plasma levels of hVEGF-A_165_ were measured with enzyme-linked immunoassay (#DVE00; R&D Systems).

### Immunohistochemistry and angiogenic responses

Avidin-biotin-HRP system was used for immunohistochemical analyses of 5 µm-thick paraffin-embedded sections fixed with 4% paraformaldehyde in 7.5% sucrose for 4 h. hVEGF-A protein was immunostained with AB-293-NA (1:500, R&D Systems), endothelium with lectin antibody (Biotonylated Griffonia (Bandeiraea) Simplicifolia Lectin I, dilution 1:100, Vector Laboratorios) and the transduction efficacy of AdLacZ was verified with anti-β-galactosidase antibody AB 1211 (1:2500, Millipore). Photographs of the sections were taken with Olympus AX70 microscope (Olympus) and AnalySIS software was used for the quantification of the amount of transduced cells (Soft Imagining System GmbH). Capillary and sinusoid areas were measured from five microscopic fields of lectin immunostained sections at X400 magnification within each animal by using AnalySIS software.

### Statistical analyses

Data scatters were visually inspected to follow the Gaussian distribution and parametric statistical tests were chosen for further analyses. Statistical analyses were done by one-way ANOVA using Bonferroni’s Multiple Comparison test as a post-test when comparing multiple groups and by Student’s t-test when comparing two groups. All results are presented as mean ± standard deviation. P < 0.05 was considered statistically significant. The following symbols are used in the figures: *P < 0.05, **P < 0.01, ***P < 0.001.

## Electronic supplementary material

Supplementary Table S1
