# Supplementary material for: High Plasma Lipid Levels Reduce Efficacy of Adenovirus-Mediated Gene Therapy
Source: Sci Rep. 2017 Mar 24;7:386. doi: 10.1038/s41598-017-00376-5 (PMC5428218; doi:10.1038/s41598-017-00376-5)
Supplement: Supplementary file 1 — Supplementary Table S1 [file 41598_2017_376_MOESM1_ESM.pdf]

Supplementary Information

High Plasma Lipid Levels Reduce Efficacy of Adenovirus-Mediated Gene Therapy

Authors: A.M. Kivelä, J. Huusko, E. Gurzeler, A. Laine, M.H. Dijkstra, G. Dragneva, C.B.F. Andersen, S.K. Moestrup, S. Ylä-Herttuala

	**C57Bl/6j control**	**C57Bl/6j + RAP 30min before Ad**		**C57Bl/6j + RAP-Ad preincubation**
**Plasma hVEGF-A expression after gene transfer (ng/ml)**	189±89.3	554.3±228.4 (high expression group)	4.24±2.8 (low expression group)	2449.9±1075.0

Supplementary Table S1. Ad preincubation with RAP increases transduction efficiency and plasma transgene levels via possible complex formation. RAP injection 30 min before Ad administration either increased or decreased plasma hVEGF-A levels compared to control mice, most probably depending on the interaction preferences of RAP with either Ad or LRP. After Ad preincubation with RAP, plasma transgene levels were robustly increased, most likely due to direct interaction of Ad and RAP leading to increased virus uptake via LRP and enhanced transduction efficiency.
